# Ergonomic T-Handle for Minimally Invasive Surgical Instruments

**Published:** 2016-05-16

**Authors:** J Parekh, DET Shepherd, DWL Hukins, N Maffulli

**Affiliations:** 1 Department of Mechanical Engineering, School of Engineering, University of Birmingham, Birmingham, UK; 2 Department of Trauma and Orthopaedic Surgery, University of Salerno Faculty of Medicine, Surgery and Dentistry, Salerno, Italy; 3 Centre of Sports and Exercise Medicine, Queen Mary University of London, Barts and The London School of Medicine and Dentistry, London, UK

**Keywords:** Ergonomic, handles, minimally invasive surgery, surgical instruments

## Abstract

A T-handle has been designed to be used for minimally invasive implantation of a dynamic hip screw to repair fractures of the proximal femur. It is capable of being used in two actions: (i) push and hold (while using an angle guide) and (ii) application of torque when using the insertion wrench and lag screw tap. The T-handle can be held in a power or precision grip. It is suitable for either single (sterilised by γ-irradiation) or multiple (sterilised by autoclaving) use. The principles developed here are applicable to handles for a wide range of surgical instruments.

## I. INTRODUCTION

The handle of a surgical instrument is the point of contact between the surgeon and the patient. It is essential that it is comfortable to use, and should aid effective control of the surgical instrument. The requirement for good design is increasingly important with the increased popularity of laparoscopic or minimally invasive surgery (MIS) in which the surgeon operates through small incisions. This has led to the design of new instruments for MIS [1,2] and of new handles for MIS instruments [3–5]. However, there are no published design studies on ergonomic design of T-handles suitable for use in MIS. In particular, the T-handle was designed for implantation of a dynamic hip screw (DHS) to aid repair of fractures of the proximal femur [6]. A T-handle is used in the conventional surgical procedure [7] and was the starting point for designing an improved ergonomic handle for MIS. T-handles are commonly used in other surgical procedures.

## II. DESIGN REQUIREMENTS

The design requirements are listed below.

1. The handle should be comfortable and convenient to use, in the manner described in points 2 and 3 below, by surgeons with a wide range of hand sizes.
2. To successfully implant a DHS, the handle should be capable of being used in two actions: (i) push and hold (while using an angle guide) and (ii) application of torque when using the insertion wrench and lag screw tap [7].
3. The handle should be capable of being gripped in more than one way to suit the surgeon’s preference. Methods of gripping can be classified as: (i) a power or cylinder grip, in which the handle is held by the fingers and thumb in the palm of the hand and (ii) a precision or ball grip, where the handle is pinched between the fingers and the thumb [8].
4. The handle should have no sharp edges that might, for example, tear a surgical glove.
5. The handle should be suitable for single or multiple use applications, and therefore the materials used in its construction should be capable of being cleaned and sterilised according to appropriate standards [9–11].
6. The handle should comply with the standards and regulations for surgical instruments [12].
7. The handle should be capable of being colour-coded for easy identification during use at different stages of the surgical procedure.

## III. DESIGN ATTRIBUTES AND CONCEPTS

### Handle length

A minimum handle length of 125 mm, with an extra 12.5 mm when gloves are worn, has been recommended for a precision grip handle [13]. The minimum length for a power grip is 100 mm, with 125 mm being considered more comfortable. The handle also needs to be sufficiently long to prevent it digging into the palm of the holder. The recommended lengths comfortably exceed the hand breadth (metacarpal) of 95 mm and the hand breadth (across thumb) of 114 mm for 95% of men [14].

### Cross-section

The cross-sectional shape of a handle depends on the intended use of an instrument. A rectangular cross-section gives more purchase but cylindrical handles are more comfortable to hold [15]. A diameter that is too large will lead to muscle fatigue because it is difficult to grip; a diameter that is too small will lead to high local pressures on the tissues of the hand [13]. The literature recommends a diameter in the range 30-50 mm [14,15]. The lower end of the range is recommended for flexibility and dexterity; the upper end is recommended to generate maximum torque [16].

### Overall shape

The main principle in the ergonomic design of hand tools is to fit the tool to the hand [17]. It has been suggested that the handle should be curved with a minimum radius of curvature of about 25 mm for the surface that engages with the hand [14]. A double frustum, in which the diameter of the handle reduces on both sides of this axis of the T provides a comfortable grip [18]. Maximising grip surface area would enable the pressure to be exerted over as large an area as possible; it would also reduce shear stress on the glove surface [19].

### Material selection

To comply with design requirement 5, all materials need to withstand multiple cycles of autoclaving, for multiple use, and to be able to withstand γ-irradiation as the most convenient sterilization method for single use [20]. The handle needs to be soft to the touch, for comfort, but sufficiently strong to withstand the forces to which it will be subjected and provide a firm grip. These requirements can be met by using layers of different materials. In addition, the surface needs to be sufficiently rough to avoid slippage when gripped but not so rough as to damage the surgeon’s glove.

### Concepts

The attributes described in the previous section were taken into account to develop two concept designs that are described below.

1. Concept 1 was a double frustum incorporating finger shaping. This concept was eventually discarded because the finger shaping made it difficult to provide comfort and gripping for different tasks for a wide range of hand sizes.
2. Concept 2 was a curved cylinder that was curved beyond the ends of the cylinder to provide support for the surgeon’s thumbs. The concave surface of the cylinder was replaced by a curved rectangle to enable it to be securely gripped by fingers of different length and to enable smaller hands to grasp the handle comfortably. Since this concept was developed into the final design, it will not be described in detail in this section.

## IV. FINAL DESIGN

### Shape and dimensions

**Fig. 1.**
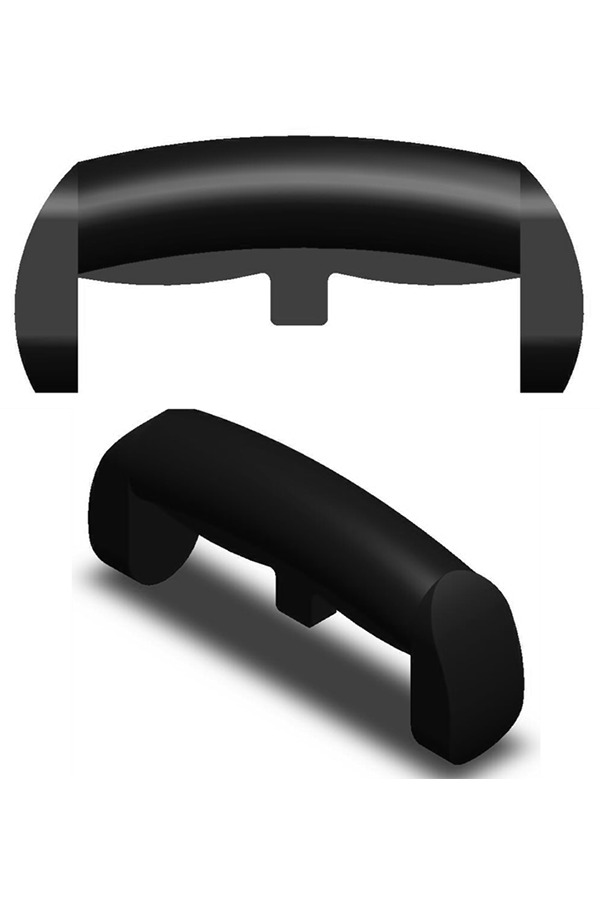
**Three-dimensional rendered image of the handle.**

**Fig. 2.**
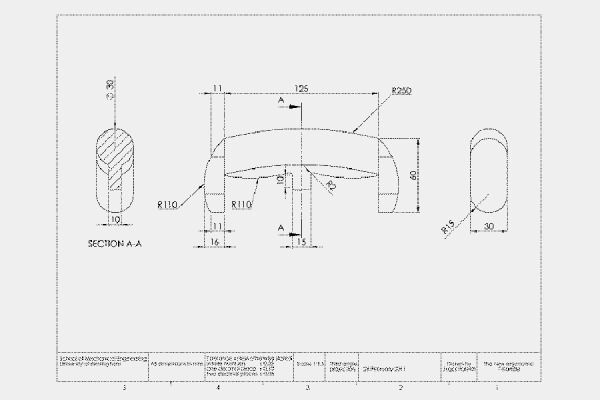
**Engineering drawing of the handle.**

Figure 1 shows the appearance of the final design; dimensions are given in Figure 2. Both drawings were produced using SolidWorks software (3DS Daussalt Systemes, Version 2010, Lowell, MA, USA). The handle is intended to be held in a power grip with some allowance for controlled rotation of the tool attached to the handle. Its length (125 mm) then allows it to extend beyond the palm of 95% of the male population (upper limit 114 mm). The additional length allows for the thumb to be placed along the shaft if required. The phalanges, at both ends, encase the fingers, preventing the tool from being dropped, even if the handle is held loosely. The width and length of the phalanges (30 mm × 60 mm) enables them to accommodate 95% of the male population (upper width limit 216 mm). The cross-section of the handle is rectangular with rounded ends (radius of curvature 30 mm). A rectangular section (width 10 mm and curved with a radius of curvature of 110 mm) enables the handle to accommodate different finger lengths and hand sizes. The combination of circular and rectangular cross-sections is intended to provide comfort and good purchase when the handle is grasped.

### Materials

Since the design was intended for multiple or single use, it would need to withstand sterilization by autoclaving (in a hospital environment) or by γ-irradiation (before delivery for single use). A curved stainless steel rod of diameter 20 mm provides the underlay for the handle. This underlay prevents excessive bending or breakage of the handle. The same grade of stainless steel is specified for this underlay as would be used to fabricate the instrument (stainless steel 316L). This is overmolded with polypropylene (PP) to give the desired shape. PP is a cheap material that can be easily processed [20] and can withstand autoclaving and γ-irradiation [21]. Then the PP layer is overmolded with thermoplastic polyolefin (TPO) elastomer to give a comfortable and soft touch. TPO allows the possibility of colour coding of surgical instruments used in a procedure and the the option of brand embossing. It can also be sterilized by autoclaving and γ-irradiation [21].

### Design verification

Failure mode and effect analysis [22,23] was used for risk analysis. The analysis was not exhaustive in that it did not include packaging, manufacture or sterilization. However, all hazards identified were addressed with effective control measures. Mechanical damage, including breakage of the handle, was identified as a hazard but considered to be low risk given the design. Damage by sterilisation was also considered low risk given the materials selection. A further possible hazard is that the handle may be unsafe to the user or uncomfortable to use. The first hazard was considered to be low risk because there are no sharp edges and the handle has been designed to avoid it slipping from the hand. An uncomfortable handle could lead to surgeon fatigue, but is low risk given that the handle was designed using anthropometric data. For a multiple use instrument, contamination of the handle with blood was identified as a hazard. This risk could be minimized by supplying appropriate cleaning and sterilisation information.

## V. CONCLUSIONS

An ergonomic T-handle has been designed for use with instruments used in MIS; in particular the specific design presented here is intended for insertion of the DHS for repair of fractures of the proximal femur. However, the principles incorporated in this design could be used in handles for a wide range of surgical procedures but especially those involving MIS where good control of the surgical instrument is especially important. The final design (shown in Figures 1 and 2) meets all the design requirements listed above. The outer polymer layer enables colour coding of instruments and other identifying information to be incorporated into the material of the handle. It can be sterilized for single or multiple use.
